# Supplementary figures and images for: A novel in vitro model to study prolonged Pseudomonas aeruginosa infection in the cystic fibrosis bronchial epithelium
Source: PLoS One. 2023 Jul 11;18(7):e0288002. doi: 10.1371/journal.pone.0288002 (PMC10335692; doi:10.1371/journal.pone.0288002)

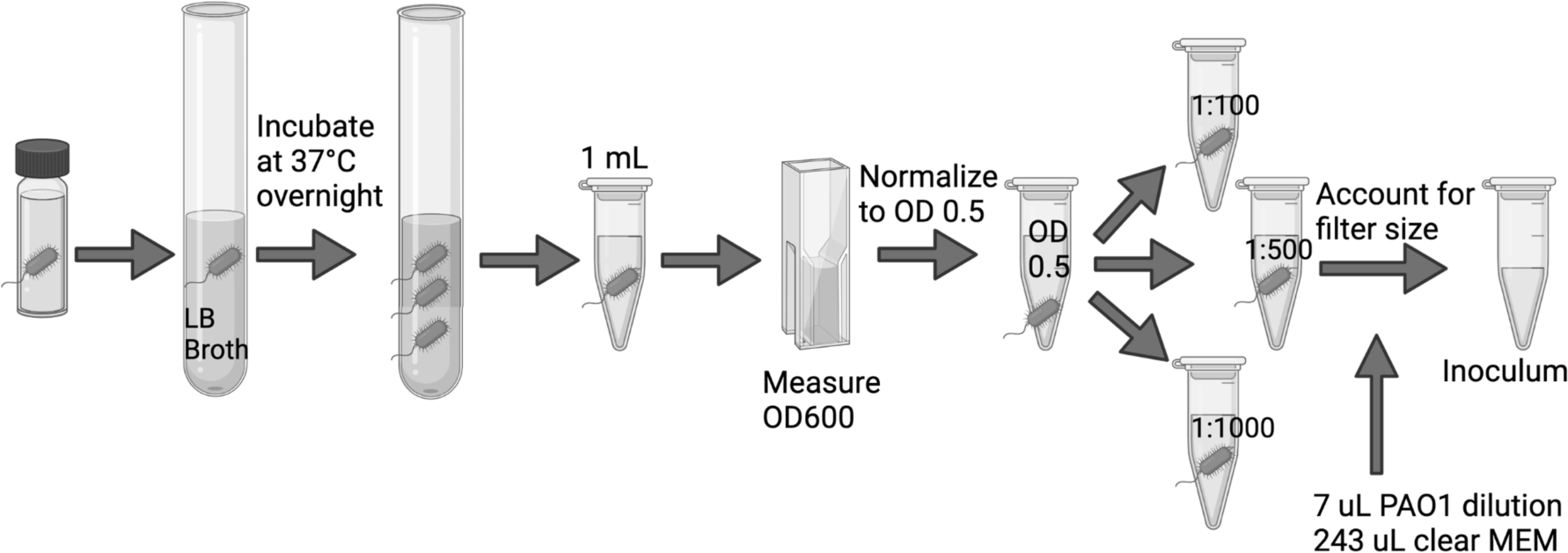

Supplement: S1 Fig — Created with BioRender.com. (TIF) [file pone.0288002.s001.tif]

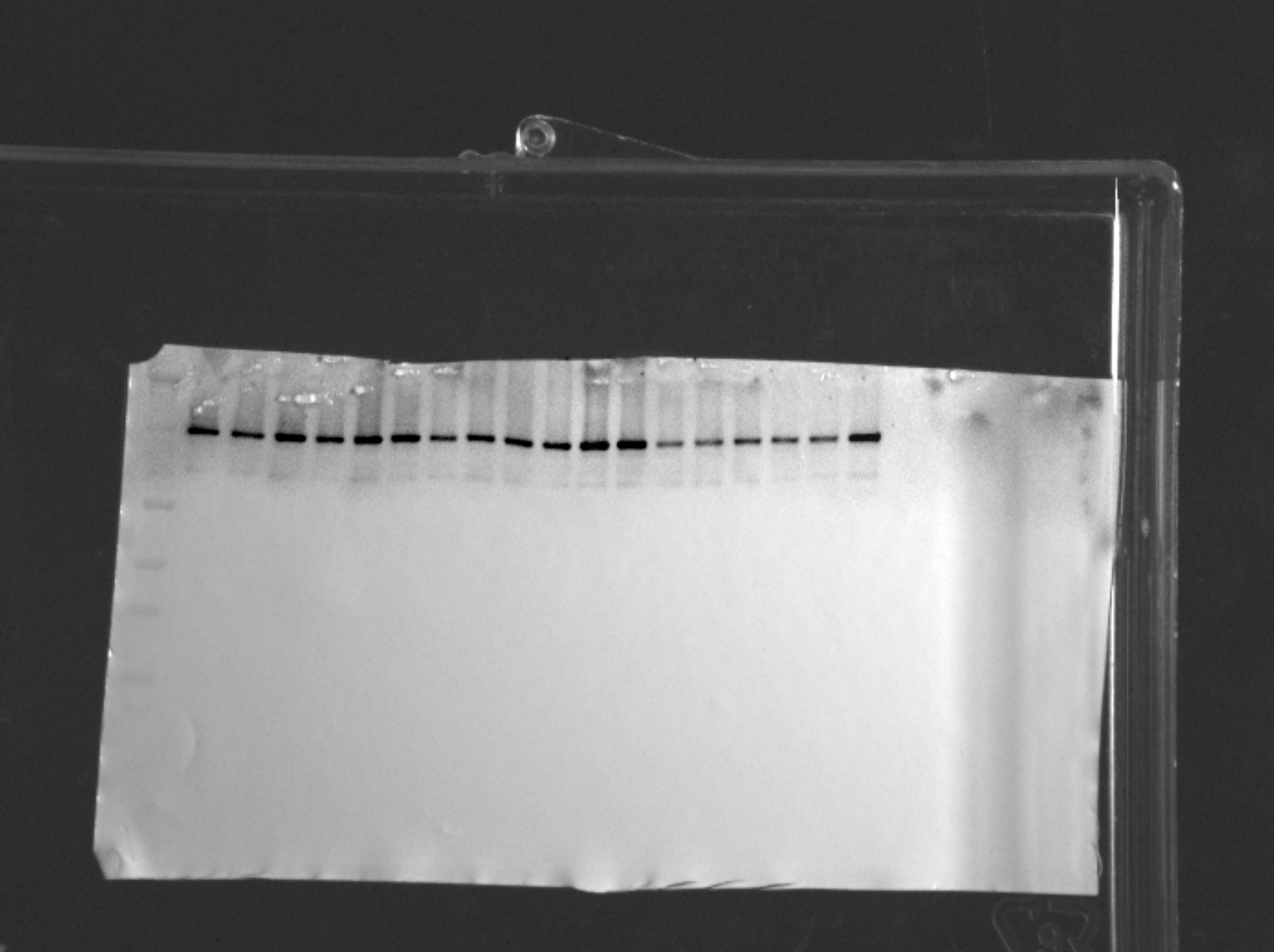

Supplement: S2 Fig — (TIF) [file pone.0288002.s002.tif]

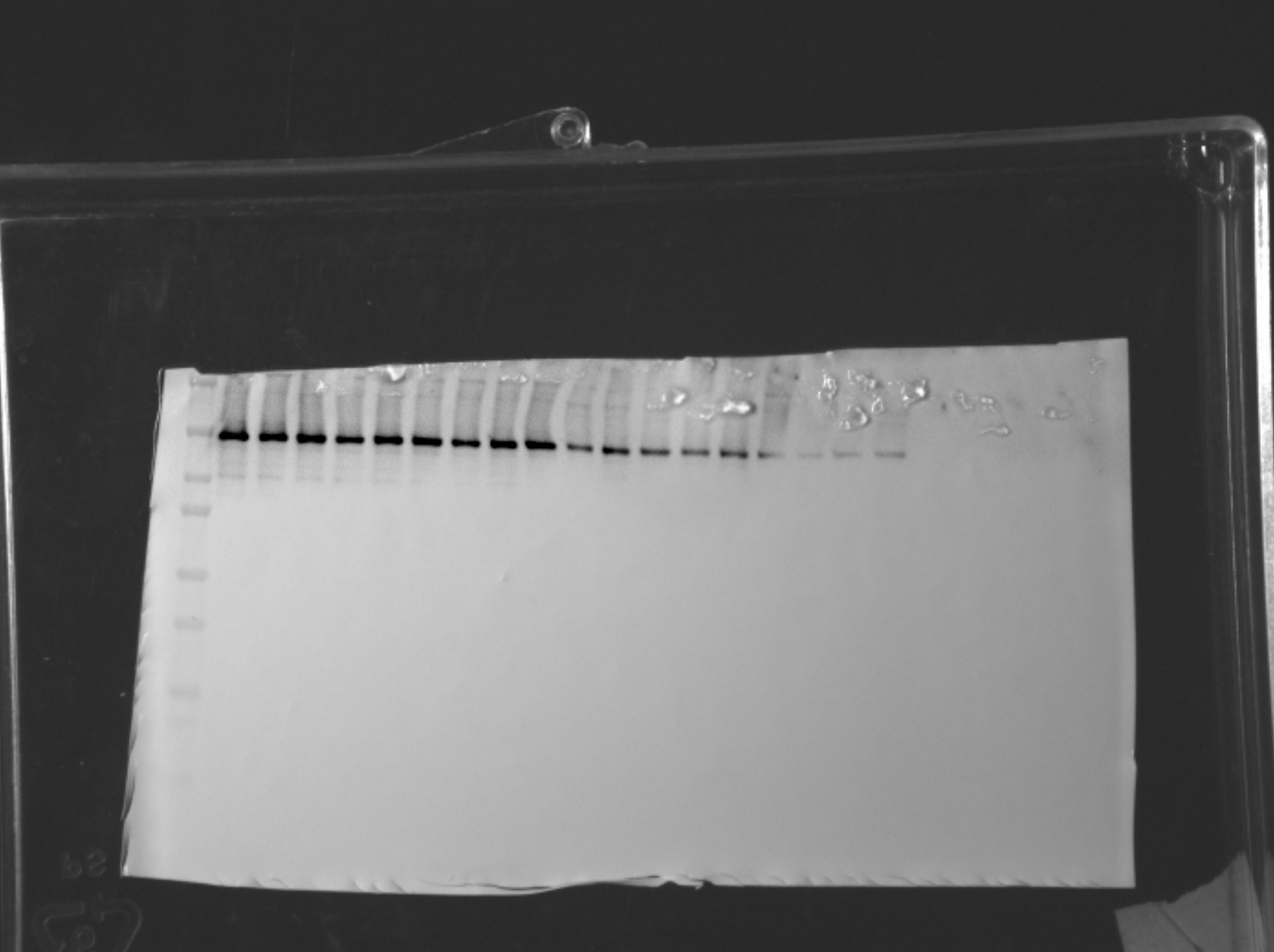

Supplement: S3 Fig — (TIF) [file pone.0288002.s003.tif]

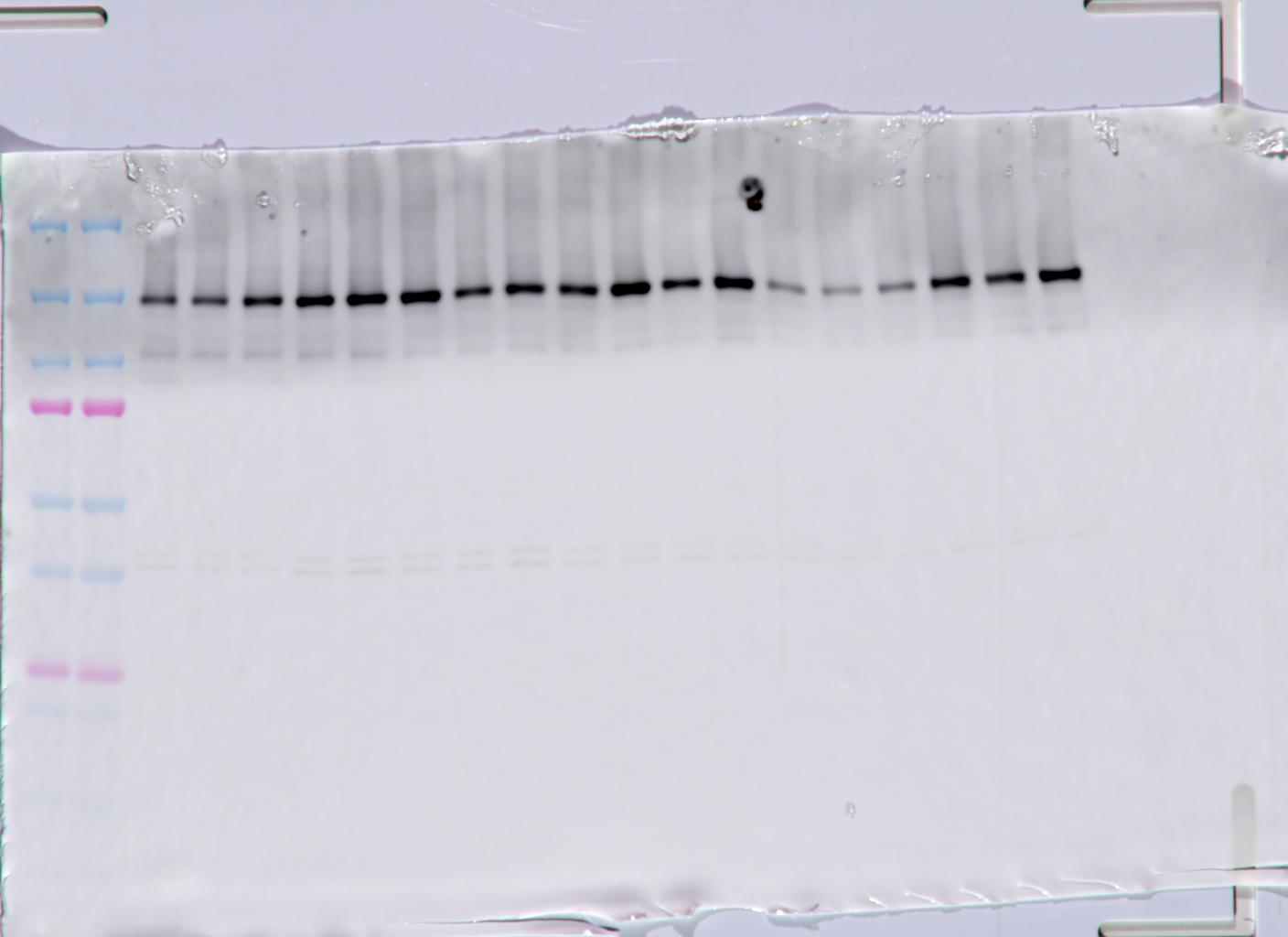

Supplement: S4 Fig — (TIF) [file pone.0288002.s004.tif]

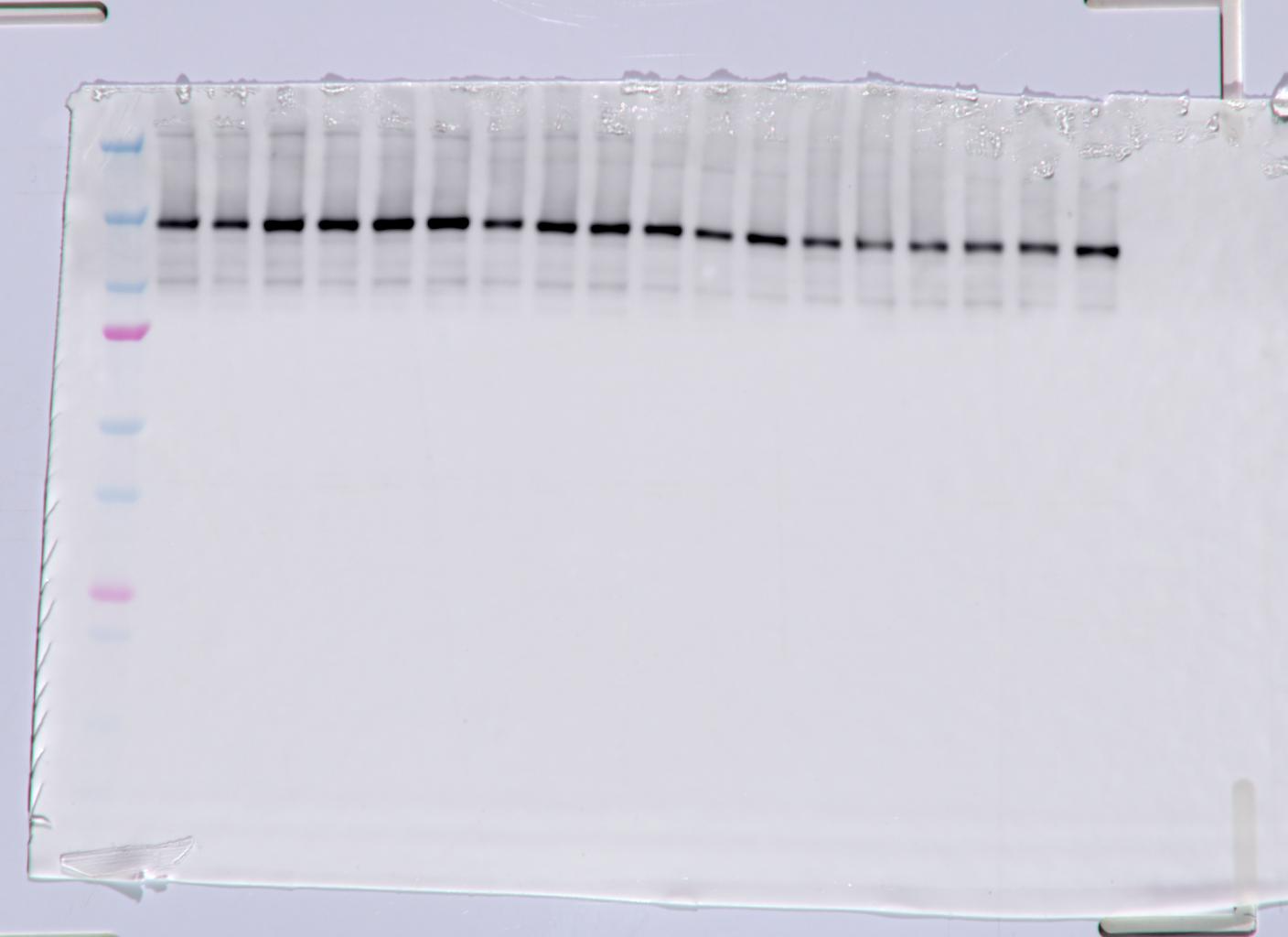

Supplement: S5 Fig — (TIF) [file pone.0288002.s005.tif]

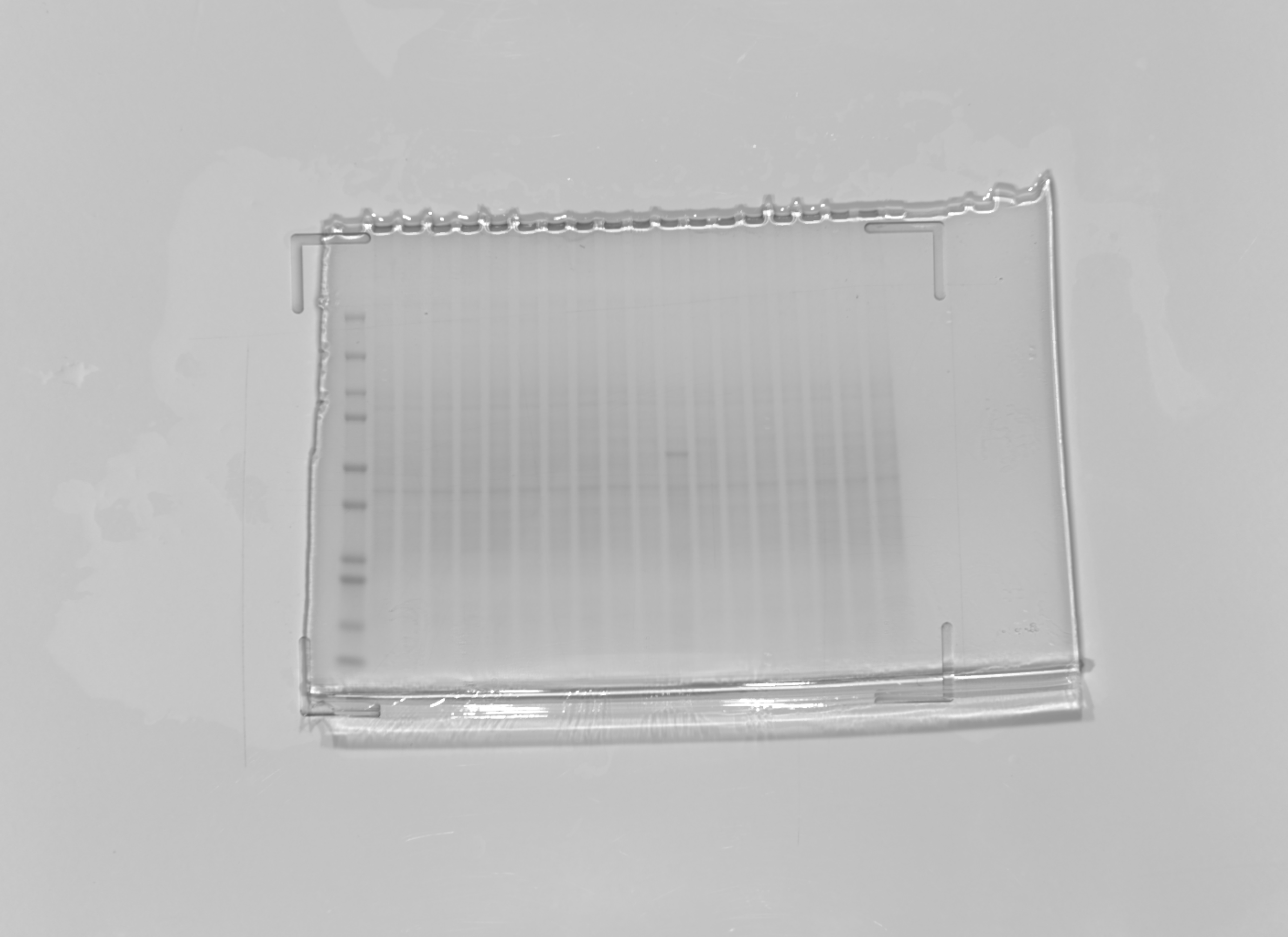

Supplement: S6 Fig — (TIF) [file pone.0288002.s006.tif]
