# Supplementary material for: A novel in vitro model to study prolonged Pseudomonas aeruginosa infection in the cystic fibrosis bronchial epithelium
Source: PLoS One. 2023 Jul 11;18(7):e0288002. doi: 10.1371/journal.pone.0288002 (PMC10335692; doi:10.1371/journal.pone.0288002)
Supplement: Attachment — Submitted filename: 04-12-2023 Response to Reviewers FINAL.docx [file pone.0288002.s007.docx]

Dear Editor and Reviewers,

We would like to thank you for the encouraging comments and constructive criticism of our submitted manuscript. This is a point-by-point response to the concerns that were addressed and our manuscript was revised accordingly and is now hopefully eligible for publication.

**Reviewer #1:** The manuscript entitled “A novel in vitro model to study prolonged Pseudomonas aeruginosa infection in the cystic fibrosis bronchial epithelium” by Meghan et al. to explore novel invitro infection model is very interesting. The manuscript is well written however, the results lack crucial data points and are not well represented. A comparison with 1-6 hours infection model needs to be shown in all data points.
Therefore, I feel the current version is not suitable for publication in PlosOne until the following major concerns are addressed.

***Answer:*** *Thank you very much for the comments. We hope that the following point-by-point responses will address your concerns sufficiently.*

Figure 1: Bright field image of the cells is not clear.

***Answer:*** *Thank you for the comment – we added further labeling and description in the revised Results section and arrows to make the images clearer.*

Figure2: CFUs for both inoculum used, and endpoint should be converted to Log CFUs for better representation. Further, the inoculum CFUs and endpoint CFUs may be plotted together for better comparison.
The authors should also include 0 hours CFUs Post infection after washing of the unbound bacteria.

***Answer:*** *Thank you for the comments. In our revised Fig. 2, CFUs have been converted to LogCFUs and both inoculum and endpoint CFUs were plotted together. In addition, we added the 1 hr timepoint to assess how much bacteria are adhering to the cells. The 0 hr timepoint is also added since it reflects that of the inoculum.*

Figure 3A: mRNA fold change for Il-8, IL-6, IL-1B, for 6 HR data is shown only for PA01. At what dilution was this data obtained? Why the authors did not include other dilutions (1:100, 1: 500, 1:1000) and the mucoid infections as shown for the 24 HR infection? Since the manuscript compares the 6 hours infection model therefore, authors must replicate the 6 HR data points as shown for 24 HR model.
Similar problem is associated with Fig 3B, C and D. Authors should include all the data points.
Fig 3C, the control cells have high levels of Il-8 while we do not see significant mRNA increase?

***Answer:*** *Thank you for those suggestions. The revised Fig. 3A, now includes mRNA fold changes of all interleukins (IL-1β, IL-8, and IL-6) for the other PA strain at the 6 h timepoint, as well as the dilution in the figure legend. As pointed out by the Reviewer, Fig. 3C, shows that control cells already high levels of IL-8, which were consistent and reproducible. We focused on developing a CF model, which exhibited the expected results. Those differences were discussed in detail in the revised results and discussion section.*

Fig 4: The western blots do not show the loading control band (GAPDH or Actin). The phosphorylated and the total proteins should be compared with the loading control in each well for densitometric analysis.
It is difficult to conclude that there is increase in phosphorylation. In fact, it looks other way round if you compare it with the untreated control data points!
Fig 4E is not clear!

***Answer:*** *Thank you for the comments and we agree with the Reviewer. We had planned to use GAPDH or beta actin as loading controls but our results demonstrated that there is actually regulation of those loading controls by PA itself, which has been shown in the literature before by Li et al, 2019 and Cowell et al, 2005. Alternatively, the densitometric analysis of the immunoblots was calculated over total protein via Coomassie stain as seen by the y-axis. We strongly believe in the effect of increased phosphorylation of PLCγ even if we used the GAPDH or beta actin, levels would be further increased.*

*See below an example of control and PA infected ALI cultures and decrease of GAPDH and beta actin levels by PA infection.*

**Reviewer #2:** Hirsch et al. has provided a well-written manuscript on Pseudomonas aeruginosa in people with CF with nice translational potential with their in vitro model. Their science is sound, and their research contribution is valuable.
I’ve attached some minor corrections, below. Other than that, I would highlight the aim of the study and the impact in the discussion. Unfortunately, I can’t seem to see the figures so I cannot comment on that.
Well done, Hirsch et al!

***Answer:*** *Thank you for your valuable assessment. In the revised manuscript, we emphasized the aim more as well as the impact in the revised abstract and discussion.*

L36: comma after tissue – *this was corrected*
L37: comma after lungs– *this was corrected*
L39: remove space between 25-34– *this was corrected*
L40: capital G for Gram– *this was corrected*
L47,49: ‘therapy’ following HEMT seems redundant? – *this was changed.*
L80: phospholipase C gamma (PLCy) – *this was corrected*
L92: remove ‘-’ (i.e., 1 h) – *this was corrected*
L107,112: 4 °C– *this was added*
L113: 60× magnification (use symbol not the letter x) – *this was corrected*
L130-131: Roche cOmplete™ Protease Inhibitor Cocktail– *this was corrected*
L132: elsewhere you’ve not cited location with company, i.e. Millipore Sigma (L131), L124: ThermoFischer. Please keep it consistent throughout– *thank you,* *this was modified accordingly*.
L146: were (?) performed– *this was corrected*
L147: standard error of mean (SEM) – *this was corrected*
L148: P is always capitalized and italicized– *this was corrected*
L150: use hour in subheading– *this was corrected*
L166: Air Liquid Interface (ALI) – *this was corrected*
L170-171: use hours, hour in subheading– *this was corrected*
L187: tdTomato– *this was corrected*
L216: italicise ‘post hoc’– *this was corrected*
L244: 6 to 24– *this was corrected*
Throughout: hrs is an incorrect abbreviation of hours. Instead of hr / hrs, use h– *this was corrected*.
