# Supplementary material for: A novel in vitro model to study prolonged Pseudomonas aeruginosa infection in the cystic fibrosis bronchial epithelium
Source: PLoS One. 2023 Jul 11;18(7):e0288002. doi: 10.1371/journal.pone.0288002 (PMC10335692; doi:10.1371/journal.pone.0288002)
Supplement: Attachment — Submitted filename: 05-04-23 Response to Editor Final.docx [file pone.0288002.s008.docx]

Dear Editor,

We would like to thank you for the constructive criticism of our submitted manuscript. This is a point-by-point response to the concerns that were addressed, and our manuscript was revised accordingly and is now hopefully eligible for publication.

Line 130: gene glyceraldehyde 3-phosphate dehydrogenase (GAPDH) was used as reference in QRT PCR, while the authors dispute the use of GAPDH and B actin as controls in the western blot. Please use another internal control for fold change calculations. How was the fold change calculated? Please describe in methods.

*Answer:* *Thank you for your comments. The literature (Li et al, 2019 and Cowell et al, 2005) stating that B-actin and GAPDH are affected by PA only demonstrates this at the protein level, which was also observed by us, but we did not see the same regulation on mRNA level. We though greatly appreciate the editor’s suggestions and performed additional experiments using another internal control. The data was analyzed using both GAPDH and a ribosomal control (RPLPO) and we found no significant difference between the CTRLs or either PA strain (Fig. 1, rebuttal letter). Using both internal controls independently demonstrated significant increases between the uninfected and infected cells as seen in the manuscript in Figure 3A. Furthermore, as requested by the editor, the methods have been revised and updated to reflect the fold change calculations.*

Did the authors quantify the total proteins in the lysates before loading? The authors should use equal amounts of proteins in each well for normalization. How were the results compared in densitometer analysis?

Fig. 1: Normalization to either GAPDH or RPLPO as internal controls both showed significant increases in IL-8 mRNA levels by PA infection to a similar extent.

*Answer: The total proteins were quantified before loading using a Bradford assay and 40 μg of protein was loaded for each sample into their respective well. The results were compared by dividing p-PLCγ by total PLCγ and then dividing by the total protein for normalization as shown previously (Krick et al., ERJ 2018). This is reflected in the y-axis of the quantifications and the methods have been revised accordingly. Additionally, the Coomassie stain can be found in the Supporting Data section, which was also used to assure comparable protein loading.*

The authors used various dilutions for infections (1:100, 1:500, 1:1000). For better comparison, the authors should have optimized it with real numbers of CFUs.

*Answer: The real numbers of CFUs are found in Figure 2A and are described in the results section pertaining to Figure 2 as well.*

Figures provided are very blurred and not clear. Therefore, not suitable for publication at this stage.
Please provide clear Images in fig 1A and 2B.
All figures provided should be clear and distinct. Error bar font sizes are too big and placed irregularly.

*Answer: We apologize that those pictures did not show up clearer in the PDF version. This has been corrected.*
