# Supplementary material for: A novel in vitro model to study prolonged Pseudomonas aeruginosa infection in the cystic fibrosis bronchial epithelium
Source: PLoS One. 2023 Jul 11;18(7):e0288002. doi: 10.1371/journal.pone.0288002 (PMC10335692; doi:10.1371/journal.pone.0288002)
Supplement: Attachment — Submitted filename: 06-01-2023 Response to Editor.docx [file pone.0288002.s009.docx]

Dear Editor,

We would like to thank you for the constructive criticism of our submitted manuscript. This is a point-by-point response to the new concerns that were addressed, and our manuscript was revised accordingly and is now hopefully eligible for publication.

Description of Fig 2 legends is superficial. Fig 2A and its description is not clear. Please improve the bar diagram and the error bars specifically the round filled dots (Reduce the size).

*Answer: Thank you for those suggestions. The round filled dots have been reduced in size and the figure legend description was updated to include more details and improve the clarity.*

Is the inoculum same to infect at different time points (1 hr, 6h and 24 hr)?
If yes then it should not be repeated for every time points instead it should be mentioned as zero time point (zero hours, 1 hours, 6 hours 24 hours). The term endpoints should be then removed!

*Answer: The inoculum is the same at the different time points. These values are now seen as the 0 h timepoint. Additionally, the terminology inoculum vs. endpoints has been revised according to the reviewer’s suggestions in the revised manuscript.*

The inoculum and the endpoint nomenclature is not clear in the experimental description.
(Dilutions of the initial inoculum may be mention as 102, 0.5 x 103 , 103 etc.
All figure legends should be described in detail including the statistical analysis.

*Answer: The inoculum and endpoint nomenclature has been updated. All figure legends have been revised as well. Statistics were run for Fig 1 or Fig 2 and all statistical analyses are now described in the figure legends. Additionally, the statistical analysis section has been updated as well.*

Additionally, in reviewing our reference list we found one reference that was incorrect. Reference #13 was originally Sanchez, M. et al, *Cells*, 2022. It now reads Sermet-Gaudelus I., et al, *Eur Respir Rev*, 2013. We apologize for this incorrect reference. It has now been corrected. No references cited are retracted articles. Thank you.
